# Signalling crosstalk in FGF2-mediated protection of endothelial cells from HIV-gp120

**DOI:** 10.1186/1471-2202-6-8

**Published:** 2005-02-02

**Authors:** Dianne Langford, Rosemary Hurford, Makoto Hashimoto, Murat Digicaylioglu, Eliezer Masliah

**Affiliations:** 1 Department of Pathology, University of California, San Diego, La Jolla, CA, USA; 2 Department of Neurosciences, University of California, San Diego, La Jolla, CA, USA; 3 The Burnham Institute Center for Neuroscience and Aging, La Jolla, CA, USA

## Abstract

**Background:**

The blood brain barrier (BBB) is the first line of defence of the central nervous system (CNS) against circulating pathogens, such as HIV. The cytotoxic HIV protein, gp120, damages endothelial cells of the BBB, thereby compromising its integrity, which may lead to migration of HIV-infected cells into the brain. Fibroblast growth factor 2 (FGF2), produced primarily by astrocytes, promotes endothelial cell fitness and angiogenesis. We hypothesized that treatment of human umbilical vein endothelial cells (HUVEC) with FGF2 would protect the cells from gp120-mediated toxicity via endothelial cell survival signalling.

**Results:**

Exposure of HUVEC to gp120 resulted in dose- and time-dependent cell death; whereas, pre-treatment of endothelial cells with FGF2 protected cells from gp120 angiotoxicity. Treatment of HUVEC with FGF2 resulted in dose- and time-dependent activation of the extracellular regulated kinase (ERK), with moderate effects on phosphoinositol 3 kinase (PI3K) and protein kinase B (PKB), also known as AKT, but no effects on glycogen synthase kinase 3 (GSK3β) activity. Using pharmacological approaches, gene transfer and kinase activity assays, we show that FGF2-mediated angioprotection against gp120 toxicity is regulated by crosstalk among the ERK, PI3K-AKT and PKC signalling pathways.

**Conclusions:**

Taken together, these results suggest that FGF2 may play a significant role in maintaining the integrity of the BBB during the progress of HIV associated cerebral endothelial cell damage.

## Background

Maintenance of blood brain barrier (BBB) integrity is critical to prevent the passage of potentially harmful factors, such as pathogens or toxins into the brain. During the progression of central nervous system (CNS) infectious disease, pathogens might gain access to the brain by compromising the integrity of the BBB. In the course of AIDS, HIV enters the brain at early stages, disrupting the components of the BBB resulting in a chronic state of inflammation known as HIV encephalitis (HIVE) [1,2]. HIVE is characterized by the presence of HIV-infected microglia and / or macrophages in the brain, the formation of multinucleated giant cells and microglial nodules, astrogliosis and myelin pallor, the combined effects of which could result in cognitive impairment [3]. Because endothelial cells of the BBB provide the first point of contact between blood-borne viral products and the brain, they provide the front line of defence against viral entry into the CNS. Alterations in signalling between components of the BBB with either HIV proteins or factors produced in response to HIV infection, such as cytokines and chemokines, may disrupt BBB integrity, resulting in a compromise that could promote transmigration of activated monocytes or HIV infected cells into the brain. Toxic viral products released by HIV-infected cells such as, gp120, Tat or Nef, together with cytokines and chemokines from activated monocytes, can act to increase BBB permeability [4-8].

Cell-free gp120 is found in the serum of HIV infected patients and crosses the BBB by absorptive endocytosis [9] and has been detected in the perivascular regions of the brain [10]. Gp120 is toxic to uninfected cells such as cerebral endothelial cells [8], and induces numerous signalling alterations in glial cells leading to indirect neuronal dysfunction and death [11,12]. Huang et al. have shown that gp120 promotes apoptosis in human umbilical vascular endothelial cells (HUVEC) by acting through CXCR4 and CCR5 chemokine receptors to increase activation of protein kinase (PKC) [13,14]. Furthermore, these studies show that the toxic effects of gp120 were blocked by PKC antagonists, sphingosine, phorbol esters and fibroblast growth factor 2 (FGF2) [13].

While viral products and inflammatory response proteins may damage components of the BBB, other factors, such as growth factors, may work to preserve BBB integrity through maintaining endothelial cell fitness. In this context, FGF2 is of particular interest for several reasons. First, FGF2 is produced primarily by astrocytes that are in proximity to cerebral endothelial cells in the blood brain barrier [15]. Among the known astrocyte-derived growth factors, only FGF2 mimics the signalling actions of astrocytes to the BBB [15,16]. Second, of the four FGF receptors (FGFR), FGFR1 is mainly expressed on neurons and endothelial cells while FGFR2 and FGFR3 are found on glial cells [17-19]. FGF2, which binds to FGFR1, exhibits a wide range of angiotrophic effects [15,16] and promotes the survival of cortical and hippocampal neurons [15,16,20-22]. Third, FGF2 signals through FGFR1 and activates phosphoinositol 3 kinase (PI3K), protein kinase C (PKC), extracellular regulated kinase (ERK), and p38 pathways [23-25]. Both ERK and p38 belong to the mitogen-activated protein kinase (MAPK) signalling pathways and have been shown to be involved in regulating endothelial cell survival [15,16]. FGF2 protection of HUVEC from gp120 is proposed to occur by preventing the gp120-mediated increase in PKC activity [13], however, protective signalling mechanisms directly induced by FGF2 have not been addressed. Therefore, we investigated the signalling pathways involved in FGF2-mediated protection against gp120 toxicity in HUVEC. Our studies indicate that FGF2 protects endothelial cells from gp120-mediated toxicity by crosstalk among several signalling pathways downstream of the tyrosine kinase FGFR. These pathways include, the ERK, PI3K/AKT and PKC signalling cascades. Likewise, other studies have suggested that signalling pathways that inhibit cell death (e.g., p38, MAPK/ERK) and survival pathways (e.g., AKT/PKB) may represent the next investigational step in inhibition of HIV-related CNS toxicity [26]. In this context, FGF2-mediated signalling may play an important role in maintaining BBB integrity during HIV trafficking into the brain and/or cell-free gp120 interactions with cerebral endothelial cells.

## Results

### FGF2 protects endothelial cells from gp120-mediated toxicity

**Figure 1:**
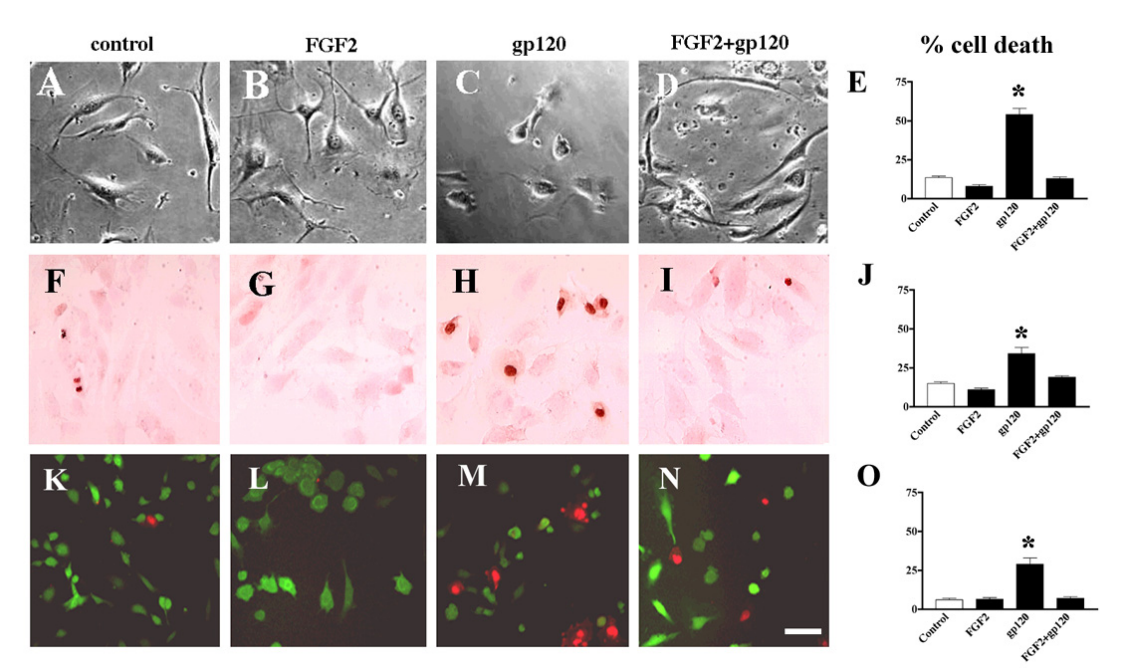
**Cell viability assays for FGF2 protection of HUVEC against gp120-mediated toxicity **(A-D) Phase contrast, (F-I) TUNEL staining, and (K-O) fluorescent staining of HUVEC. Panels A, F, and K show images of untreated HUVEC control; B, G, and L show HUVEC treated for 24 h with FGF2 (20 ng/ml); panels C, H, and M show HUVEC treated for 24 h with gp120 (25 ng/ml) and panels D, I, and N show HUVEC pre-treated with FGF2 for 24 h before a 24 h exposure to gp120. Panels E, J and O show the percentage of cell death as determined by Trypan blue exclusion, percentage of DNA fragmentation by TUNEL and FA/PI staining, respectively. Results shown in panels E, J and O represent the average of three separate experiments performed in triplicate. * = P < 0.05 by One-Way ANOVA with post-hoc Dunnett's when compared to control. Bar = 20 microns.

**Figure 2:**
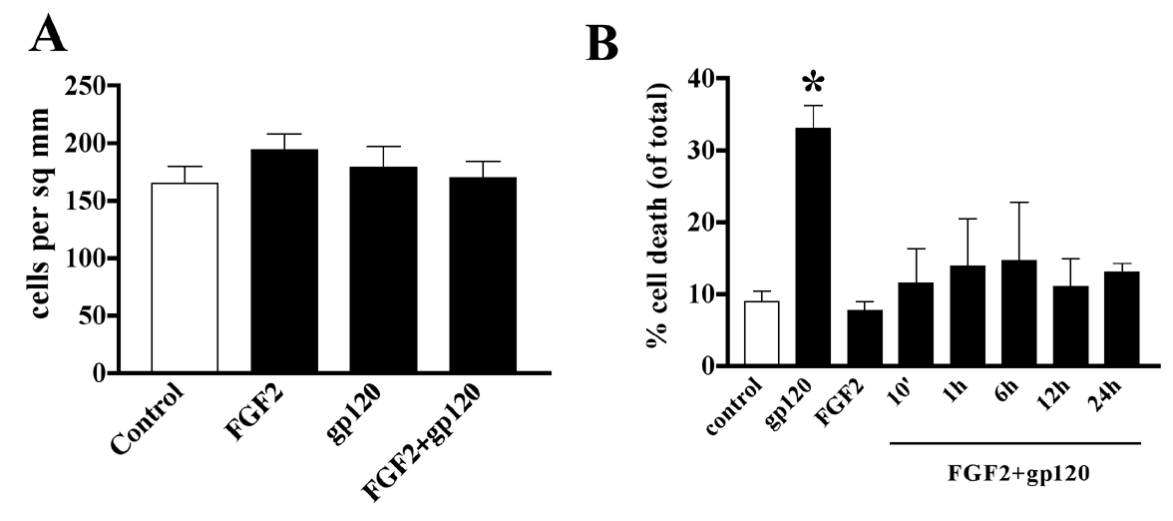
**Effects of FGF2 and gp120 on cell proliferation and viability **A) Density of proliferating HUVEC exposed to FGF2 and/or gp120. B) Cell viability of cells exposed to gp120 after pre-treating HUVEC with FGF2 for different lengths of time. Cell viability was measured 24 h after gp120 addition. * = P < 0.05 by One-Way ANOVA with post-hoc Dunnett's when compared to control.

Consistent with previous reports [13,14,27,28], our results showed that gp120 (25 ng/ml) increased cell death of HUVEC above control by approximately 27.5% (average of results from all viability assays) after 24 h exposure (Fig. 1) as determined by Trypan Blue Exclusion, TUNEL, and FA/PI staining (Fig. 1E, J, O, respectively). However, cells pre-treated with FGF2 (20 ng/ml) for 24 h and then exposed to gp120 displayed essentially the same percentage of cell death as untreated control cells (Fig. 1D, E, I, J, N, O). Although FGF2 treatment of HUVEC most likely improved overall cell fitness [15,16], no significant differences in the total numbers of cells (Fig. 2A) or in cell viability were observed between control and FGF2 treated cultures (Fig. 1E, J, O and 2B). Furthermore, time course experiments indicated that simultaneous treatment (data not shown) or pre-treatment with FGF2 up to 24 h was effective at protecting cells from gp120 toxicity (Fig. 2B). These results indicate that FGF2 is protective against gp120-mediated toxicity in HUVEC.

### FGF2 activates ERK in HUVEC

**Figure 3:**
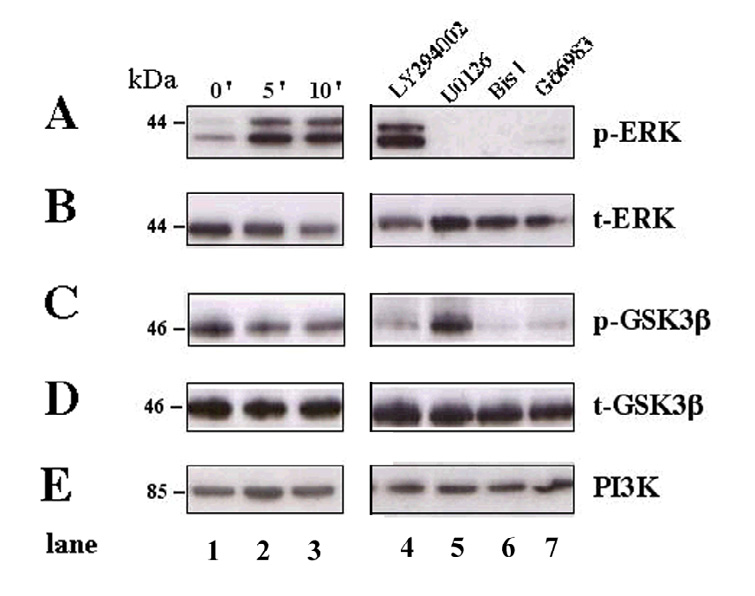
**Effects of inhibitors on FGF2-mediated ERK activation A) **Western blot (WB) reacted with anti-phospho-ERK after treatment with FGF2 for 0, 5, 10 min (lanes 1–3) and after inhibitors LY294002 (to block PI3K), U0126 (to block MEK), and Bis I and Gö6983 (to block PKC) (lanes 4–7, respectively). **B) **WB reacted with anti-total ERK antibody. **(C) **WB reacted with anti-phospho-GSK3β. **D) **WB reacted with anti-GSK3β. **E) **WB reacted with anti-PI3K antibody. The same WB was used in each experiment after stripping, reblocking and incubating with new antibodies for the given protein.

To explore mechanisms involved in the angio-protective effects of FGF2 against gp120, we first investigated FGF2-stimulated signalling mechanisms that are involved in cell survival pathways. The binding of FGF2 to its receptor (FGFR1) induces several signalling cascades, such as MAPK-mediated ERK activation and AKT-mediated GSK3β inactivation, both of which regulate cell survival. We first determined the effects of FGF2 stimulation on phosphorylation of ERK and GSK3β in time course experiments in HUVEC (Fig. 3, lanes 1–3). Western blot analysis showed that HUVEC treated with FGF2 (20 ng/ml) resulted in maximum ERK phosphorylation 5–10 min after stimulation (Fig. 3A, lanes 1–3), followed by a progressive decrease reaching undetectable levels at 60 min (data not shown), with no effect on levels of total ERK (Fig. 3B, lanes 1–3). Neither GSK3β (Fig. 3C) nor PKC (data not shown) phosphorylation was affected by FGF2 treatment.

To test the specificity of FGF2 on signalling, HUVEC were exposed to pharmacological inhibitors for PI3K (LY294002), ERK (U0126), and PKC (Bis I and Gö6983) for 30 min prior to FGF2 treatment (Fig. 3A, lanes 4–7, respectively). ERK phosphorylation was inhibited by blocking ERK and PKC (Fig. 3, lanes 5–7). Interestingly, blocking the PI3K/AKT/GSK3β pathway resulted in a dramatic increase in ERK phosphorylation (Fig. 3A, lane 4). Neither FGF2 nor inhibitors affected levels of total ERK (Fig. 3B). With regard to GSK3β, blocking PI3K with LY294002 and PKC with Bis I or Gö6983 also inhibited GSK3β phosphorylation (Fig. 3C, lanes 4, 6, 7), albeit to a lesser degree. Treatment with the ERK inhibitor U0126 increased GSK3β phosphorylation (Fig. 3C, lane 5). Neither FGF2 nor inhibitors affected total levels of PI3K or GSK3β (Fig. 3D, E). These inhibitor studies suggest that FGF2 signalling involves crosstalk between PI3K/AKT/GSK3β and ERK that is possibly mediated by PKC (Fig. 3A, lane 4 and 3C, lane 5).

**Figure 4:**
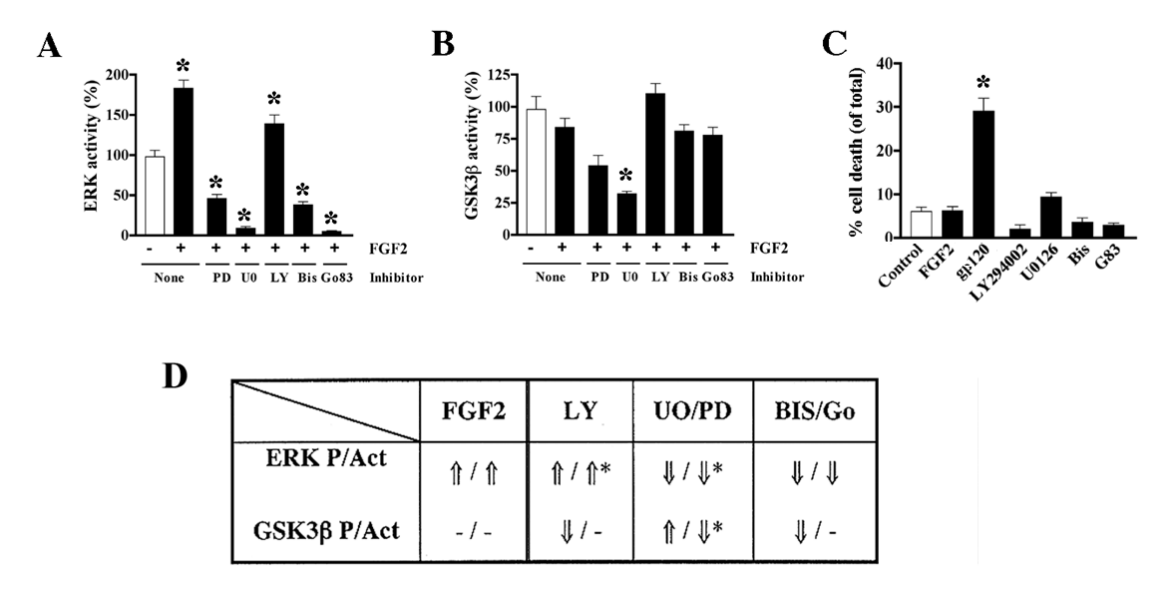
**Effects of inhibitors on FGF2-stimulated ERK and GSK3β activity **Immunocomplex kinase assays for **(A) **ERK and **(B) **GSK3β activity without FGF2 treatment, or after inhibition with PD98059, U0126, LY29004, Bisindolymaleimide I or Gö6983 followed by FGF2 treatment. **(C) **Inhibitor treatment alone. (**D**) Summary of changes in phosphorylation (P) versus changes in activity (Act) of ERK and GSK3β. ⇑ Indicates an increase, ⇓ indicates a decrease, – indicates no change. * = P < 0.05 by One-Way ANOVA with post-hoc Dunnett's when compared to control.

To further confirm that these changes in kinase signalling are mediated by FGF2, immuno-complex kinase assays were performed (Fig. 4A, B). As indicated by an astrisk (*) in Fig. 4A, lane 2, FGF2 treatment increased ERK activity significantly above levels observed in un-treated control cells (Fig. 4A). Likewise, and as shown in Figure 3, FGF2-mediated ERK activity was significantly greater than control in the presence of the PI3K inhibitor LY294002 (Fig. 4A). The ERK inhibitors PD and U0126, and the PKC inhibitors Bis I and Gö6983 significantly blocked FGF2-mediated ERK activity (Fig. 4A, D) as shown in Figure 3. Conversely, FGF2 alone or in the presence of the inhibitors LY294002, Bis I and Gö6983 had minimal effects on GSK3β activity (Fig 4B, D). However, the ERK inhibitor U0126 significantly decreased GSK3β activity (Fig. 4B). PD98059 also decreased GSK3β activity although at statistically insignificant levels (Fig. 4B). Cell viability was not significantly affected by FGF2 or inhibitor treatments (Fig. 4C) ensuring that effects of inhibitors on kinase activity were not due to cell death.

Taken together these data show that FGF2 activates ERK signalling in HUVEC but has little effect on GSK3β activity unless FGF2-mediated ERK phosphorylation is blocked. Furthermore, independently of FGF2, PI3K/AKT and PKC signalling is necessary for GSK3β phosphorylation. However, once GSK3β is phosphorylated, the kinase activity of GSK3β is independent of PI3K/AKT and PKC downstream signalling. On the other hand, GSK3β phosphorylation is influenced, to some degree, by FGF2-mediated ERK phosphorylation since blocking ERK phosphorylation results in a significant increase in the phosphorylation of GSK3β. Likewise, the kinase activity of GSK3β also appears to require ERK phosphorylation for maximal activation. In summary, the FGF2-mediated kinase activity of ERK and GSK3β appears to involve crosstalk between these pathways and possibly PKC. The potential roles of ERK and GSK3β phosphorylation and activity in FGF2-mediated protection from gp120 were investigated.

### FGF2 angioprotection in HUVEC against gp120 toxicity is mediated, in part, by ERK signalling

**Figure 5:**
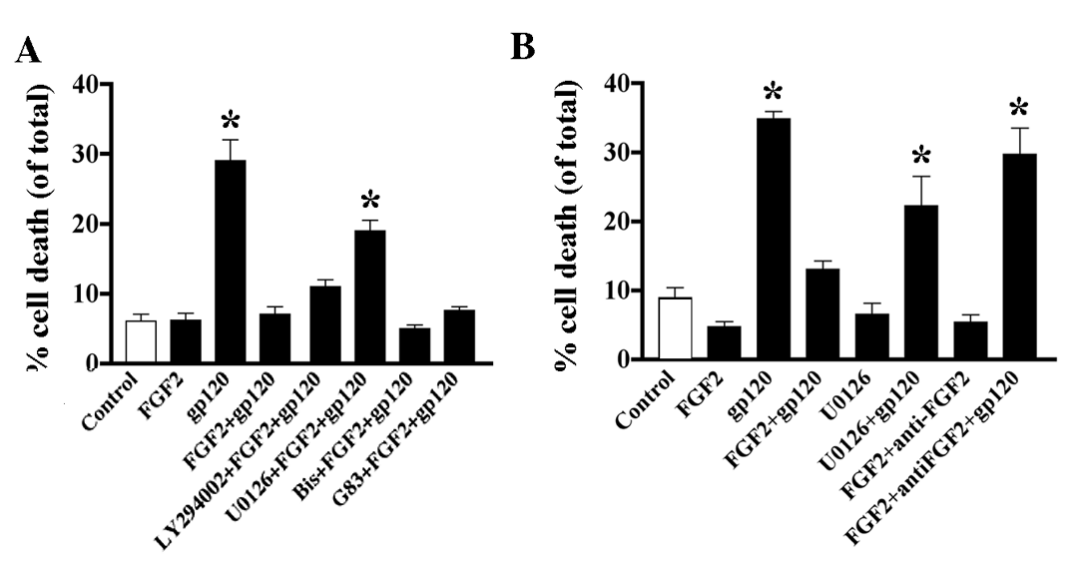
**Viability assays indicate that ERK activation is required for FGF2 protection against gp120 (A**) Trypan Blue Exclusion assay for cell survival after 30 min of treatment with inhibitors LY294002, U0126, Bis I, or Gö6983 followed by treatment with FGF2 and/or gp120. **(B) **Trypan Blue Exclusion assay for cell survival after 30 min of treatment with the MEK inhibitor U0126 alone, and followed by exposure to gp120 and/or FGF2 for 24 h. In cells treated with FGF2 and anti-FGF2, HUVEC were exposed to anti-FGF2 antibody for 1 h followed by treatment with FGF2 alone or in combination with gp120. * Indicates a significant difference from control. * = P < 0.05 by One-Way ANOVA with post-hoc Dunnett's when compared to control.

To investigate the potential role of ERK and PI3K/AKT/GSK3β signalling in FGF2-mediated angioprotection against gp120, HUVEC were treated with LY294002, U0126, Bis I, or Gö6983 for 30 min prior to FGF2 and gp120 exposure (Fig. 5). Results from cell toxicity assays determined by Trypan blue exclusion (Fig. 5A), support our previous data (Fig. 1) showing that exposure to gp120 alone significantly increased cell death above control and FGF2 treated cells; whereas, cells pre-treated with FGF2 before exposure to gp120 were protected (Fig. 5). The protective effects of FGF2 against gp120 were significantly blocked by U0126, which inhibits MEK to block ERK phosphorylation, (Fig. 5A). Blocking PI3K with LY 294002 partially blocked FGF2 protection, although at levels insignificant from control. FGF2 protection from gp120 was not affected by blocking PKC with Bis I or Gö6983 (Fig. 5A). Treating cells with U0126 to block ERK phosphorylation, and gp120 in the absence of FGF2 resulted in significant cell death compared to untreated cells (Fig. 5B). Moreover, pre-incubation of FGF2 with anti-FGF2 antibody completely neutralized FGF2-mediated angioprotection against gp120 (Fig. 5B). These results indicate that ERK phosphorylation is significantly involved in FGF2-mediated angioprotection from gp120. PI3K/AKT/GSK3β signalling is partially involved in FGF2 protection from gp120; whereas, PKC signalling in the presence of FGF2 is not necessary for protection from gp120. These results suggest that FGF2 protects endothelial cells from gp120 largely by ERK stimulation with a partial contribution by GSK3β phosphorylation.

**Figure 6:**
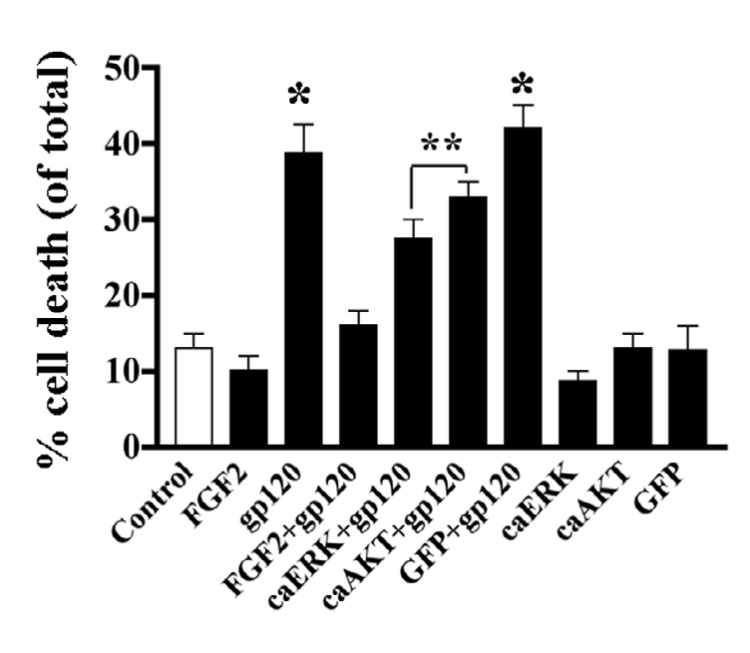
**Gene transfer of constitutively active ERK and AKT protect cells from gp120 toxicity (A) **After gene transfer of constitutively active (ca) ERK or ca AKT, HUVEC were exposed to gp120, FGF2 or a combination of both and cell viability was assayed via Trypan Blue Exclusion. Controls consisted of ca ERK, ca AKT and GFP gene transfer without further treatment. * indicates a significant difference from control. ** indicates a significant difference from *. * = P < 0.05 by One-Way ANOVA with post-hoc Dunnett's when compared to control. ** = P < 0.05 by One-Way ANOVA with post-hoc Tukey-Kramer when compared between experimental groups.

To further confirm the contribution of these signalling pathways in FGF2 protection against gp120, HUVEC infected with caERK or caAKT were exposed to gp120 and assayed for cell viability. As expected, endothelial cells infected with caERK and exposed to gp120 were significantly protected from gp120 toxicity (Fig. 6). caAKT conveyed only partial protection from gp120 toxicity, less than either caERK or FGF2 treatment (Fig. 6). In control experiments where HUVEC were infected with GFP adenovirus, no protective effects against gp120 were observed (Fig. 6). Furthermore, none of the adenoviral constructs alone promoted significant cell toxicity (Fig. 6). In agreement with our previous data, these results suggest that ERK activation plays a significant role in protection of endothelial cells from gp120, and AKT/GSK3β is also be involved.

**Figure 7:**
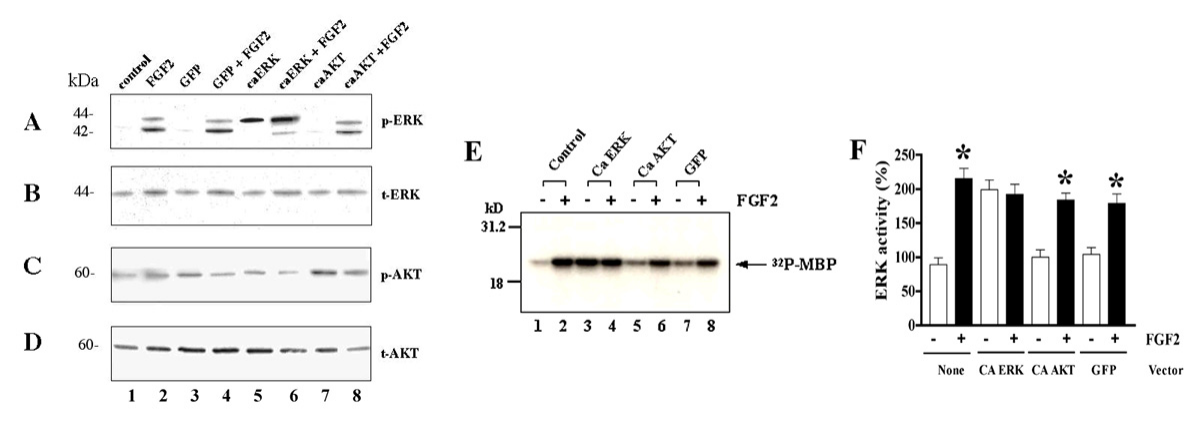
**Effects of gene transfer of constitutively active ERK, AKT or GFP phosphorylation (A-D) **Western Blot of HUVEC after treatment with FGF2, infected with the GFP, ca ERK or ca AKT adenoviral constructs, or treated with FGF2 and infected with adenoviral constructs. The same Western blot was used for all antibodies after stripping and rehybridizing. A) Reacted with anti-phospho ERK antibody **(B) **reacted with anti-total ERK antibody. **(C) **Reacted with anti-phospho AKT antibody. **(D) **Reacted with anti-total AKT antibody. **(E) **Immuno-complex assay showing changes in ERK activity with or without FGF2 treatment in HUVEC with caERK, caAKT or GFP. **(F) **Quantification of ERK activity levels in HUVEC +/- FGF2 treatment with caERK, caAKT or GFP with the PhosphorImager as described in the Materials and Methods. * indicates a significant difference from control. * = P < 0.05 by One-Way ANOVA with post-hoc Dunnett's when compared to control.

To confirm that the gene transfer approach resulted in ERK and AKT phosphorylation and kinase activation, Western blot (Fig. 7A–D) and immuno-complex assays (Fig. 7E, F) were performed. ERK kinase activity was detected using an antibody that recognizes only the phosphorylated form of ERK1/2. Consistent with our previous experiments (Fig. 3A), FGF2 stimulation resulted in an increase of both ERK1 (44 kDa) and ERK2 (42 kDa) phosphorylation (Fig. 7A). Levels of FGF2-mediated phosphorylation of ERK2 were greater than ERK1 (Fig. 7A, lane 2). Infection with the GFP adenoviral construct alone had no effect on ERK1/2 phosphorylation (Fig. 7A, lane 3). In contrast, infection with caERK resulted in a significant increase in ERK1 phosphorylation with no effect on ERK2 (Fig. 7A, lane 5). FGF2 treatment in combination with caERK induced high levels of ERK1 phosphorylation with only moderate increases in ERK2 phosphorylation (Fig. 7A, lane 6). These results indicate that FGF2 stimulation results in phosphorylation of mainly ERK2; whereas gene transfer of caERK or the combination of FGF2 and caERK mainly increased ERK1 phosphorylation. Importantly, total ERK activity levels were similar in caERK with or without FGF2 (Fig. 7E and 7F). Moreover, the level of protection conveyed by FGF2 alone was similar to protection by caERK or caERK plus FGF2.

On the other hand, caAKT alone had no effect on ERK1/2 phosphorylation (Fig. 7A, lane 7), whereas, FGF2 treatment in combination with caAKT (Fig. 7A, lane 8) had similar effects on ERK1/2 phosphorylation as observed with FGF2 (lane 2) alone or with GFP and FGF2 (lane 4). Levels of total ERK were not affected by FGF2, GFP, caERK or caAKT (Fig. 7B). Infection of HUVEC with caAKT resulted in a slight increase in baseline levels of AKT phosphorylation (Fig. 7C, lane 7). Levels of total AKT were not affected by FGF2, GFP, caERK, or caAKT (Fig. 7D). Consistent with Western blot analyses, immunocomplex assays show that caERK and/or FGF2 increased levels of ERK activity (Fig. 7E, lanes 2–4, 6 and 7F), whereas neither caAKT nor GFP resulted in increased ERK activity in the absence of FGF2 (Fig. 7E lanes 5, 7 and Fig. 7F).

Results from inhibitor studies (Fig. 5) and gene transfer experiments (Fig. 6) suggest that both ERK and PI3K/AKT (albeit to a lesser degree) are involved in FGF2-mediated protection against gp120 toxicity. Furthermore, blocking the ERK-mediated pathway results in an increase in GSK3β phosphorylation and vice versa: blocking the AKT/GSK3β pathway after FGF2 stimulation results in an increase in ERK phosphorylation. These results suggest that when endothelial cells are exposed to gp120, FGF2 may mediate protection that involves crosstalk between the ERK and PI3K pathways (Fig. 3A and 3C, and Fig. 6). Moreover, inhibitor studies suggest PKC may be involved in this signalling convergence, but a direct role of PKC in FGF2 protection against gp120 is unclear.

### PKC may be involved in crosstalk between ERK and AKT signalling pathways during FGF2 protection from gp120

Our studies using pharmacological inhibitors suggest that PKC may be involved in a crosstalk mechanism observed between the ERK and AKT/GSK3β pathways in FGF2 signalling. For example, when HUVEC were exposed to PKC inhibitors Bis I and Gö6983 prior to FGF2 treatment, ERK phosphorylation was inhibited to below baseline levels, showing that FGF2-mediated ERK phosphorylation is at least in part influenced by PKC phosphorylation (Fig. 3A, lanes 6 and 7). Likewise, PKC inhibitors partially inhibited GSK3β phosphorylation after FGF2 stimulation (Fig. 3C, lanes 6 and 7). Furthermore, since Huang et al. have shown that total PKC phosphorylation increases with gp120 treatment in HUVEC and that FGF2 is protective [13], we explored the possibility that similar crosstalk might be involved in the FGF2-mediated protection from gp120. To address these signalling events, we determined which signalling pathways were initiated by FGF2 and which were initiated by gp120.

**Figure 8:**
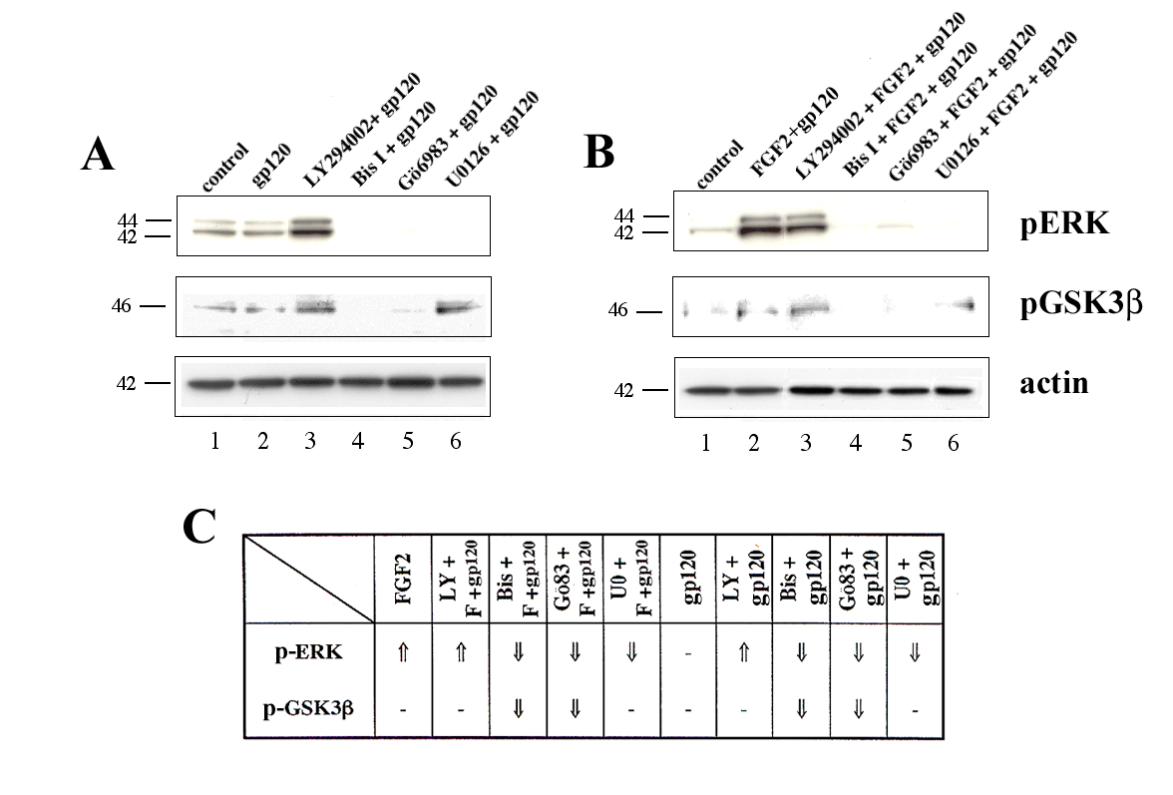
**Effects of FGF2, gp120, and inhibitors on ERK and GSK3β phosphorylation (A, B) **Western blots showing ERK and GSK3β phosphorylation with **(A) **gp120 alone (lane 2), and with inhibitors (lanes 3–6), **(B) **FGF2 and gp120 (lane 2), and FGF2 with inhibitors and gp120 (lanes 3–6). **(C) **Table summarizing data from western blots **(A **and **B) **showing changes in phosphorylation of ERK and GSK3β. ⇑ Indicates and increase, ⇓ indicates a decrease, – indicates no change.

To differentiate the effects of gp120 on ERK, GSK3β and PKC phosphorylation from those obtained in Fig. 3 where FGF2 alone was utilized, we treated endothelial cells with 1) gp120 alone (Fig. 8A, C), 2) gp120 in combination with inhibitors (Fig. 8A, C), and 3) inhibitors, FGF2 and gp120 (Fig. 8B, C). Treatment of endothelial cells with gp120 alone (Fig. 8A, lane 2, 8C) or with inhibitors alone (data not shown) did not change levels of ERK phosphorylation. However, when endothelial cells were treated with LY204002 and then exposed to gp120 for 30 min, a significant increase in ERK phosphorylation was observed (Fig. 8A, lane 3, 8C). Furthermore, in the presence of both FGF2 and gp120 and the inhibitor LY294002, ERK phosphorylation also increased (Fig. 8B, lane 3, 8C). Interestingly, in the presence of the PKC inhibitor that includes inhibition of the ζ isoform (Gö6983), ERK phosphorylation is returned to approximately control levels (Fig. 8A–B, lane 5, 8C). On the other hand, inhibition of the classic isoforms of PKC, α, β and γ with Bis I almost completely blocks ERK phosphorylation in the presence of FGF2 and gp120 (Fig. 8B, lane 4), as does inhibition of ERK phosphorylation with U0126 (Fig. 8B, lane 6). These results suggest that PKC signalling may be involved in FGF2-stimulated ERK phosphorylation that protects against gp120. Treatment of HUVEC with gp120 alone, or with gp120 and inhibitors to block ERK, or PI3K/AKT/GSK3β had little effect on GSK3β phosphorylation (Fig. 8A, lanes 1–3, 6, 8C); whereas, blocking PKC decreased levels of GSK3β phosphorylation (Fig. 8A, lanes 4 and 5). Likewise, treatment of HUVEC with FGF2 alone or with FGF2, gp120 and inhibitors to block PI3K/AKT/GSK3β or ERK had little effect on GSK3β phosphorylation (Fig. 8B, lanes 1–3, 6, 8C); whereas, blocking PKC decreased levels of GSK3β phosphorylation (Fig. 8B, lanes 4 and 5, 8C). In summary, in the presence of FGF2 and inhibitors for FGFR and PI3K/AKT/GSK3β, ERK phosphorylation increases (Fig. 3A). However, in the presence of FGF2 or FGF2 and inhibitors for PKC or ERK, ERK phosphorylation decreases (Fig 8A, B, C). Likewise, PKC inhibitors almost completely abolish GSK3β phosphorylation in the presence of gp120, independently of FGF2 stimulation (Fig. 8B, C). Together, these findings point to PKC involvement with FGF2 stimulated signalling in HUVEC during challenge with gp120, however further experimentation is needed to confirm any role of PKC in FGF2-mediated protection from gp120.

## Discussion

**Figure 9:**
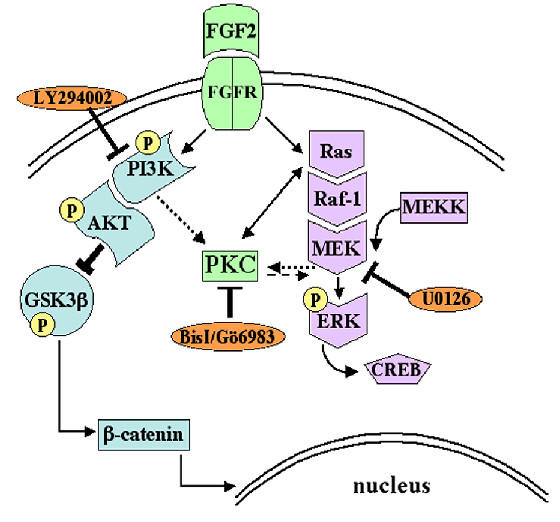
**Diagrammatic representation of signalling pathways that may be involved in FGF2-mediated protection from gp120 **As indicated by the diagram and as described by the data, it is possible that the crosstalk between the PI3K/AKT/GSK3β and ERK pathways is mediated in part by PKC. PKC signalling also is reported to occur directly with PI3K and upstream of Ras. Furthermore, direct interaction between MEK and PKC downstream of Ras is reported. Likewise, ERK2 (p42) is reported to signal to Raf-1 via a positive feedback mechanism. The points of action of the inhibitors LY290042, U0126, Bisindolymaleimide I, and Gö6983 are also shown.

The present study is the first to show that FGF2 protects HUVEC against the toxic effects of gp120 via crosstalk of the ERK-PI3K/AKT pathways (Fig. 9). Consistent with these finding, FGF2 has been shown to protect endothelial cells from oxidative stress [29] and radiation [30,31]. These studies suggest that PKC is involved in protection against ultra-violet radiation, since blocking PKC abrogates FGF2-mediated protection [31]. Similarly, a recent study showed that FGF2 also protected endothelial cells from gp120-mediated toxicity that was induced by dysregulation of PKC activity to promote apoptosis [13,28]; however, the pathways by which FGF2 protected endothelial cells from gp120 remained unclear and may be represented by independent mechanisms. Therefore, our study focused on signalling pathways involved in angioprotection upon exposure to gp120. gp120 has been reported to dysregulate PKC signalling but also to induce ERK phosphorylation in several systems by different pathways [32-34]. Likewise, our studies suggest that gp120 and FGF2 signalling in HUVEC may, in some aspects, overlap and involve primarily ERK and to a lesser extent AKT/GSK3β signalling. In this context, when HUVEC were treated with the ERK inhibitor U0126, then exposed to gp120, a significant increase in cell death above control was observed; however, the amount of cell death observed under these conditions was less than that observed in cells treated with gp120 alone. In HUVEC, PKC phosphorylation does not change when stimulated with FGF2 and PKC does not appear to be directly involved in FGF2-mediated protection from gp120 since inhibitors of this pathway had no effect on angioprotection. However, previous studies have shown that PCK may play a role in the MAPK signalling cascade, through upstream crosstalk with Ras (Figure 9) [35,36]. Moreover, in the presence of gp120 with or without FGF2, both ERK and PKC inhibitors completely block ERK phosphorylation, suggesting that while PKC is involved in ERK phosphorylation, the protective properties of ERK are not dependent on PKC. In support of these conclusions, the current study shows that inhibition of ERK, and to a lesser degree PI3K/AKT, blocks FGF2-mediated protection from gp120. Our data suggest that FGF2 signalling via ERK-PI3K/AKT crosstalk is responsible for protection of endothelial cells from gp120. Other mechanisms that could contribute to FGF2-mediated protection against gp120 may include, but are not limited to, interaction of FGF2 with heparin sulfate receptors and/or stimulation of alternative pathways not involving ERK [37].

Consistent with these findings, FGF2 protects cardiac myocytes from inducible nitric oxide synthetase induced apoptosis by the ERK signalling pathway [38], and in neuronal cells FGF2-mediated ERK activation is essential for survival signalling [39]. Our studies provide evidence for the first time that FGF2-mediated protection of endothelial cells against gp120 toxicity largely occurs through an ERK-dependent pathway. Our data also suggest crosstalk between the PI3K/AKT and ERK pathways, since blocking PI3K resulted in a significant increase in ERK phosphorylation in FGF2 treated endothelial cells. Likewise, blocking ERK caused an increase in phosphorylation of GSK3β, which is directly downstream of PI3K/AKT signalling. In this context, it is possible that upon stimulation by growth factors such as FGF2, endothelial cells utilize several signalling cascades that are capable of crosstalk to promote cell fitness and survival, as suggested by studies involving vascular endothelial growth factor (VEGF) signalling in the presence or absence of serum [40]. In these studies, it was shown that crosstalk between the AKT and p38 pathways may regulate cell survival during serum withdrawal and VEGF stimulation [40]. Our studies also point toward signalling crosstalk during FGF2 protection from gp120. Crosstalk between PI3K and p38 was shown to be mediated by MAPK kinase kinase (MEKK3) in VEGF signalling [40]. Likewise, in FGF2 signalling, crosstalk between PI3K/AKT and ERK might be mediated by PKC [41]. This is consistent with previous studies showing that in VEGF-stimulated endothelial cells, inhibition of PI3K resulted in an increase in the phosphorylation of ERK1/2 and p38 phosphorylation [42]. Together with the findings in this study, these reports emphasize the importance of different signalling pathways communicating to regulate intracellular signal transduction in endothelial cell survival [43,44].

The observations reported in this study have potential importance to the maintenance of BBB integrity in host response during HIV infection. FGF2 is produced by astrocytes in close proximity to endothelial cells of the BBB and functions to improve cell fitness and barrier integrity. In *in vitro *models of the BBB, FGF2 treatment of endothelial cells mimics the effects of astrocyte co-culture by improving tight junction integrity [15]. Numerous studies have shown that disruption of this key component in the BBB is central to HIV infection of the CNS and is a hallmark of HIVE [45]. This is particularly important during HIV trafficking into the CNS because endothelial cells of the BBB are the first neural cells to come in contact with HIV infected cells or HIV products. Regulation among signalling crosstalk in endothelial cells by FGF2 is important since these are the cells of the BBB that first encounter HIV-infected and/or activated cells and HIV products such as gp120. Migration of HIV-infected and/or activated cells into the brain is largely regulated by endothelial cell integrity. During the progression of HIVE, activated and HIV-infected monocytes produce cytokines and chemokines and release HIV products that act in concert to compromise the integrity of the BBB [46]. This triggers a series of signalling events that may result in the alteration of tight junction proteins, such as zona occludins, thereby promoting migration of HIV-infected cells into the brain parenchyma [45,47,48]. Alternatively, astroglial cells that are also an important component of the BBB might produce trophic factors such as FGF2 in response to endothelial cell distress in attempts to maintain BBB integrity. Among them, factors produced by damaged endothelial cells, including tissue factor, can induce the early growth response-1 gene (Egr-1) transcription factor in astrocytes that in turn directs expression of FGF2 [49].

## Conclusions

In summary, the present study shows that FGF2 is protective against gp120 toxicity via crosstalk of ERK-PI3K/AKT signalling pathways during compensatory signalling. This finding is important for understanding the pathogenesis of HIVE because factors produced by components of the BBB, such as FGF2 by astrocytes, in response to toxins such as HIV-gp120 may be responsible in part for angio-protection of endothelial cells of brain microvasculature.

## Methods

### Cell culture

HUVEC (Clonetics^®^, BioWhittaker, Inc., Walkersville, MD) were grown in complete media (endothelial basal medium [EBM] supplemented with bovine brain extract (12 μg/ml), human epithelial growth factor (10 ng/ml), hydrocortisone (1 μg/ml), GA-1000 (Gentamicin and Amphotericin B, 1 μg/ml) (Clonetics) and 20% fetal bovine serum (Irvine Scientific, Irvine, CA). Complete growth media were changed to minimal media (EBM, GA-1000, 1% serum, Clonetics) for 24 h prior to treatments. HUVEC were chosen because previous studies have characterized this cell line with regard to FGF2 mediated signaling responses and much of the work conducted in the present study complements and builds on data from these studies [13,27]. Furthermore, HUVEC mimic numerous characteristics of cerebral endothelial cells. Both short-term signaling events and long-term viability of HUVEC were addressed after treatment with a combination of inhibitors, FGF2, and gp120, or with each component alone, as described below.

### HUVEC treatments to determine viability

For viability assays, HUVEC were treated with either 20 ng/ml FGF2, (Calbiochem, La Jolla, CA) or full-length recombinant HIV-1_BaL _gp120 (25 ng/ml) NIH Research and Reagent Program, Rockville, MD and Bartels-Mardx, Carlsbad, CA) for 30 min, 1 h, 6 h, 12 h and 24 h. Recombinant HIV-1_BaL _used in these experiments is a macrophage trophic virus and binds to CD4 and signals via CCR5. For protection assays, HUVEC were treated either simultaneously with FGF2 and gp120 or pre-treated with FGF2 for 30 min, 1 h, 6 h, 12 h and 24 h before the addition of 25 ng/ml gp120. HUVEC were harvested 24 h after the addition of gp120 for viability assays.

### Viability assays

For trypan blue exclusion assays, HUVEC were rinsed with warm PBS, harvested, collected by gentle centrifugation, resuspended in a PBS/ trypan blue solution (1:1, vol:vol) and counted as previously described [50].

Terminal dUTP end labeling (TUNEL) staining was carried out essentially as described previously [51,52]. Cells were grown on coverslips, rinsed with PBS and fixed with 4% paraformaldehyde for 20 min at room temperature. After rinsing with PBS, cells were permeabilized with 1% H_2_O_2 _in 1× PBS-Tween-20 for 10 min at room temperature, rinsed twice with PBS and air-dried for 2 min. TUNEL was conducted according to the manufacturer's instructions for staining (Roche Diagnostics, Indianapolis, IN) and counterstained with Eosin Y. TUNEL positive cells were detected with 3, 3' diaminobenzidine (DAB) (Sigma) and counted with a computer-aided analysis system (Quantinet 570C, Leica, Bannockburn, IL).

Cell death was also assayed by fluorescent staining with fluorescein diacetate (FA) and propidium iodide (PI) as previously described [27]. The FA (Sigma) working solution was prepared by adding 10 μl of stock FA (50 mg FA in 10 ml of acetone) to 2.5 ml PBS. The FA/PI (Sigma) cocktail was prepared by adding 1 μl of FA working solution to 300 μl of PI (1 mg PI in 50 ml PBS). After rinsing once in warm PBS, 20 μl of the FA/PI cocktail was added to cells on coverslips and incubated 15 min in the dark. Coverslips were placed cell-side up on SuperFrost slides (Fisher Scientific, Pittsburgh, PA) under anti-fading media (Vector Laboratories, Inc., Burlingame, CA) and immediately imaged with laser scanning confocal microscope (LSCM, MRC1024, Bio-RAD, Hercules, CA).

### HUVEC treatments for signalling events

Signalling events mediated by FGF2 and/or gp120 were determined via Western Blot (WB) analyses. Cells were treated with either 20 ng/ml FGF2 (Calbiochem, La Jolla, CA) or gp120 for 30 min, 1 h, 6 h, 12 h and 24 h and analyzed by WB. Additionally, HUVEC were treated with inhibitors alone. To test the effects of FGF2 stimulation or gp120 exposure on downstream signalling, prior to FGF2 treatment, cells were pre-treated with inhibitors targeting different steps in the MAPK, PKC or AKT/glycogen synthase kinase 3-beta (GSK3β) pathways. For these experiments, cells were incubated for 30 min with the: (i) 10 μM PI3K inhibitor LY294002 (Calbiochem), (ii) 10 μM PKC inhibitors Gö6983 (Calbiochem), 2 μM Bisindolymaleimide I (Calbiochem), (iii) 10 μM MEK inhibitors U0126 or 20 μm PD98059 (Calbiochem). To test the specificity of FGF2-mediated protection against gp120, HUVEC were incubated with 20-fold excess anti-FGF2 neutralizing antibody prior to the addition of 20 ng/ml FGF2. Cells were incubated in the presence of anti-FGF2 antibody and FGF2 for 24 h, then exposed to 25 ng/ml gp120 for 24 h and assayed for viability, ERK phosphorylation and kinase activity. To determine the signalling events caused by gp120, with or without FGF2 and inhibitors, the following conditions were utilized: 1) cells were treated with inhibitors for 30 min as previously described, and then exposed to 25 ng/ml for 30 min, 2) inhibitors for 30 min then gp120 for 30, 3) inhibitors for 30 min, FGF2 for 10 min and gp120 for 30 min. After treatments cells were immediately harvested for Western analyses.

### Western blot analysis in HUVEC

Briefly, after treatments, cell monolayers were harvested and solubilized in HEPES homogenization buffer (1 mM HEPES, 5 mM Benzamidine, 2 mM 2-Mercaptoethanol, 3 mM EDTA, 0.5 mM Magnesium Sulfate, 0.05% Sodium Azide, Protease Inhibitor Cocktail III and Phosphatase Inhibitor Cocktail I) (Calbiochem). Protein concentration was determined by the method of Lowry and between 10–15 μg of protein were separated by electrophoresis on 10% Bis-Tris NuPage Gels (Invitrogen, Carlsbad, CA). Samples were then electroblotted onto Immunobilon P nitrocellulose membranes (Millipore, Bedford, MA). Proteins were immunolabeled with primary antibodies against phosphoERK1/2 (1:2500) (Thr202/tyr204, mouse monoclonal phosphoERK antibody) (Cell Signalling Technology), total ERK1/2 (1:2500) (anti-mouse monoclonal ERK1/2 antibody) (Pharmigen), phosphoGSK3β (1:2500) (Ser 9, anti-rabbit polyclonal phospho-GSK3β antibody) (Cell Signalling Technology), total GSK3β (1:2500) (anti-mouse monoclonal GSK3β antibody) (Transduction Laboratories, Lexington, KY), phosphoAkt (Thr308, anti-rabbit polyclonal phosphoAKT antibody) (Calbiochem), total AKT (1:2500) (anti-rabbit polyclonal AKT antibody), (Calbiochem), anti-mouse monoclonal PI3K antibody(1:1000) (Transduction Labs), anti-rabbit phospho-PKC (pan) that detects phosphorylation of PKC isoforms α, β, δ, ε, and η (Cell Signalling Technology, Beverly, MA) and anti-rabbit actin antibody (1:1000) (Chemicon, San Diego, CA). Blots were incubated with the HRP-tagged secondary antibody, detected with the ECL reagent (DuPont NEN, Boston, MA), followed by autoradiography. As a control, HUVEC were pre-treated with one of the following pharmacological inhibitors: MTA, LY294002, Gö6983, Bisindolymaleimide I, U0126 or PD98059 for 30 min and then FGF2 and gp120 were added simultaneously. Cell viability was assayed 24 h later.

### Adenoviral constructs and transfection

Recombinant adenoviral constructs encoding constitutively active (ca) forms of ERK and AKT were prepared as previously described [53,54] (kindly provided by Dr. Kazuhiko Namikawa, Asahikawa Medical College, Asahikawa, Japan and Dr. Kenneth Walsh, Tufts University, Boston, MA, respectively). Adenovirus encoding the green fluorescent protein (GFP-Ad) as previously described [55] was used as a control to account for any effects that may be due to adenoviral infection. Briefly, for ca-ERK, cDNA fragments containing the entire coding regions for human MAP/ERK kinase 1 (MEK1) were isolated from human embryonic kidney cells (HEK293) by PCR. ca-ERK lacks the nuclear export signal (amino acids 32–51) and has glutamic acid substitutions for two phosphorylation sites, Ser^218 ^and Ser^222^, was prepared by site-directed mutagenesis and fused to the hemagglutinin tag sequence, as previously described [56]. ca-AKT, has the c-*src *myristoylation sequence fused in frame to the N-terminus of the FLAG-AKT coding sequence [54]. High titer recombinant viral stocks (10^11 ^plaque forming units) were generated in HEK293 cells and stored at -80°C. Endothelial cells were plated at approximately 50% confluency in complete media (20% serum) and grown for 24 h at 37°C, 5% CO_2_. HUVEC were changed to minimal media (1% serum) for 6 h and then half of the media was removed from each sample, pooled and stored at 37°C, 5% CO_2_. HUVEC were infected at a multiplicity of infection of 50 in pre-conditioned minimal media for 4 h, achieving a 40–50% transduction efficiency (data not shown). Minimal medium containing adenovirus was replaced with pooled pre-conditioned minimal media and cell cultures were further incubated for 48 h at 37°C and 5% CO_2_. After 48 h, cells were treated with FGF2 (10 ng/ml) for 10 min, harvested in lysis buffer, stored at -20°C, and later used for ERK and AKT kinase assays. For immunocytochemistry, cells on coverslips were blocked overnight at 4°C in 10% horse serum and 5% BSA. Coverslips for ca-ERK were then labelled overnight at 4°C with primary anti-Hemagglutinin (1:150) (Roche Diagnostics) and for ca-AKT with primary anti-FLAG (1:50) (Sigma) followed by incubation with secondary biotinylated IgG (Vector Laboratories) (1:200) for 1 h at room temperature. Hemagglutinin and FLAG proteins were detected with DAB (Sigma) and visualized by light microscopy to access HA production. Experiments were conducted at least three times to ensure reproducibility.

### Immunocomplex kinase assays

ERK and AKT Assays were performed essentially as previously described with some modifications [57]. Briefly, cells were rinsed twice with cold phosphate-buffered saline and incubated for 20 min on ice in lysis buffer (1% Triton X-100, 10 % glycerol, 50 mM HEPES, pH 7.4, 140 mM NaCl, 1 mM EDTA, 1 mM Na_3_VO_4_, 1 mM phenyl-methylsulfonylfluoride, 5 μg/ml aprotinin, 5 μg/ml leupeptin, 1 mM dithiothreitol). The cell lysates were then centrifuged for 10 min at 14,000 rpm and protein concentration was determined using the BCA reagent (Pierce, Rockford, IL). Two hundred microliters of the supernatant were pre-absorbed with a protein G-sepharose (Amersham Pharmacia Biotech, Uppsala, Sweden) for 1 h at 4°C. The pre-cleared lysates were incubated with 1 μg/sample of anti-ERK monoclonal antibody (1:50) (Pharmingen, San Diego, CA) or polyclonal anti-human AKT antibody (anti-PKB 88–100) (Calbiochem) overnight at 4°C, followed by incubation with protein G-sepharose for 2 h at 4°C. After washing twice with the lysis buffer and twice with a kinase buffer (20 mM HEPES, pH 7.2, 0.1 mM Na_3_VO_4_, 10 mM glycerophosphate, 10 mM MgCl_2_, 1 mM dithiothreitol, 0.1 mM EGTA), the immune complexes were incubated in 30 μl of the kinase buffer containing 20 μg myelin basic protein for ERK (Sigma) or 1 μg of GSK3β fusion protein (Cell Signalling, Beverly, MA) for AKT and 10 μCi of [γ-^32^P] ATP (6000 Ci/mmol, PerkinElmer, Boston, MA) for 30 min at 30 °C. Reactions were terminated by the addition of 5 μl of 500 mM EDTA and 5 mM ATP. After adding 4× Laemmili SDS sample buffer and boiling 5 min, samples were separated by 15% SDS-PAGE, followed by autoradiography. Quantification was performed with the PhosphorImager using the Image Quant software (Molecular Dynamics, Sunnyvale, CA).

### Statistical analysis

All experiments were performed in a blind code fashion. After results were obtained, the code was broken and analysis was performed by utilizing one-way analysis of variance (ANOVA) with post hoc Dunnett's or Tukey-Kramer.

## Authors' contributions

DL designed and conducted FGF2, gp120 and inhibitors experiments, Western analyses, viability assays and composed the manuscript.

RH assisted in design of, and conducted FGF2, gp120 and inhibitors experiments, Western analyses and viability assays.

MH designed and conducted activity assays and gene transfer experiments.

MD obtained adenoviral constructs, designed experimental methods for infection and analysed output measures.

EM performed light microscopy and immunocytochemical experiments and composed with DL the first draft of the manuscript.
